# Structural Features and PF4 Functions that Occur in Heparin-Induced Thrombocytopenia (HIT) Complicated by COVID-19

**DOI:** 10.3390/antib9040052

**Published:** 2020-10-10

**Authors:** Zheng Cai, Mark I. Greene, Zhiqiang Zhu, Hongtao Zhang

**Affiliations:** Departments of Pathology and Laboratory Medicine, Perelman School of Medicine, University of Pennsylvania, Philadelphia, PA 19301, USA; caizheng78@gmail.com (Z.C.); greenemarkirwin@gmail.com (M.I.G.); zhuzhuqiang@gmail.com (Z.Z.)

**Keywords:** PF4, thrombin, COVID-19, Coronavirus

## Abstract

Platelet factor 4 (PF4, CXCL4) is a small chemokine protein released by activated platelets. Although a major physiological function of PF4 is to promote blood coagulation, this cytokine is involved in innate and adaptive immunity in events when platelets are activated in response to infections. Coronavirus disease 2019 (COVID-19) patients have abnormal coagulation activities, and severe patients develop higher D-dimer levels. D-dimers are small protein products present in the blood after blood clots are degraded by fibrinolysis. To prevent clotting, heparin is often clinically used in COVID-19 patients. Some clinical procedures for the management of COVID-19 patients may include extracorporeal membrane oxygenation (ECMO) and renal replacement therapy (CRRT), which also require the use of heparin. Anti-PF4 antibodies are frequently detected in severe patients and heparin-induced thrombocytopenia (HIT) can also be observed. PF4 and its role in HIT as well as in pathologies seen in COVID-19 patients define a potential therapeutic option of using blocking antibodies in the treatment of COVID-19.

## 1. Introduction

Platelet factor 4 (PF4, CXCL4) is a small chemokine protein released by activated platelets [1]. The mature size of PF4 is 70-amino-acid protein, or 7.8 kDa. Its major physiological function is to promote blood coagulation. This function is related to PF4’s affinity for heparin and other glycosaminoglycans (GAGs) [2,3], which are long linear polysaccharides consisting of repeating disaccharide units. By neutralizing the negatively charged heparan sulfate side chains of GAGs on the surface of platelets and endothelial cells, PF4 facilitates platelet aggregation to form a thrombus.

However, PF4 has additional activities beyond simply promoting blood coagulation, and the regulation of PF4 is very complex. PF4 expression is elevated following trauma. This is perfectly understandable, as coagulation is often needed to prevent blood loss from injury. Unexpectedly, a significant amount of PF4 is also released by activated platelets in response to infection. A systematic review of the functions of platelets in infectious diseases, especially in the current Coronavirus disease 2019 (COVID-19) pandemic, will be helpful in facilitating a better understanding of the role of PF4, as well as the potential use of a PF4-blocking antibody in the management of heparin-induced thrombocytopenia (HIT).

## 2. Platelets and Viral Infection of the Lung

Although platelets have an average life span of 8 to 12 days, this special class of anucleated cell fragments is critical for many important biological processes, such as hemostasis, thrombosis, wound healing, angiogenesis, immunity, and inflammatory responses [4,5,6]. The roles of platelets in innate immunity are relevant to viral infection of the lung, an important site for platelet biogenesis. By directly imaging the lung microcirculation in mice, Liu et al. demonstrated the circulation of a large number of megakaryocytes through the lungs and the release of platelets in this organ [7]. In fact, platelets released by megakaryocytes in the lungs account for approximately 50% of total platelet production, defining the lung as a major organ for platelet biogenesis. Patients with acute influenza infection possess activated platelets that engulf influenza A H1N1 virions and release antiviral molecules (e.g., α-granules) that can destroy the virus [8]. In response to bacterial or single-stranded viral infection, platelets also form heterotypic aggregates with neutrophils.

TLR7 is a Toll-like receptor (TLR) that is expressed on platelets. TLR7 is critical for viral- engulfment and subsequent neutrophil activation. TLR7 knockout (KO) mice display reduced neutrophil-DNA release. Engulfed influenza nucleic acids are sensed by platelet-TLR7. This interaction leads to complement C3 release, which is sufficient to mediate DNA release from neutrophils. In the presence of neutrophils, platelets also secrete GM-CSF, which acts as a negative regulator of the TLR7-dependent neutrophil-DNA release [8]. It is noteworthy that rare loss-of-function variants of the X-chromosomal *TLR7* gene have been identified in four young male patients with severe COVID-19 from two families [9]. One patient failed to survive due to concurrent secondary bacterial infection. Immunological defects in type I and II interferon production is found to be associated with this *TLR7* gene mutation.

The platelet response to respiratory viruses includes granule production and release. In general platelets contain three types of granule: α-granules, dense granules, and lysosomes. α-granules are the most abundant granule type. The α-granules are composed of various chemokines, cytokines, membrane proteins, and proteases, as well as proinflammatory and anti-inflammatory mediators [3,10].

Platelets are also involved in adaptive immunity. The Fcγ receptor, FcγRIIA, is expressed on the surface of activated platelets and is responsible for the endocytosis of immunoglobulin G (IgG) coated pathogens. This has been further demonstrated by the ability of platelets from FcγRIIA transgenic mice, but not wild-type mice (which are deficient for FcγRIIA expression in platelets), to bind and endocytose IgG complexes [11]. Manne and colleagues have studied platelet gene expression and function in COVID-19 patients [12]. Differential gene expression changes have been identified in pathways related to protein ubiquitination, antigen presentation and mitochondrial dysfunction. Platelets from COVID-19 patients tend to be hyperactive, and can form significantly more circulating platelet-neutrophil, -monocyte, and -T-cell aggregates compared to healthy donors.

P-selectin is an adhesion molecule on platelets. P-selectin can bind to its receptor P-selectin glycoprotein ligand 1 (PSGL1) on myeloid and a subset of lymphocytes. The interaction between P-selectin and PSGL1 thus becomes the driving force for the adhesion, immobilization, and recruitment of leukocytes [13,14] and lymphocytes during inflammation, tissue repair and thrombotic disorders [15]. By interacting with PSGL1 on monocytes, platelet P-selectin can also induce a cross-presentation program in peripheral blood monocytes and lead to dendritic cell differentiation [16]. In COVID-19 patients, platelets have higher P-selectin expression levels both at resting and after activation [12]. This feature may account for the hyperactivity of platelets and clotting risk in COVID-19 [17,18].

## 3. Role of PF4 in Infections

During infections, activated platelets release PF4 in response to microorganisms [19,20]. PF4 contributes to the recruitment of neutrophils and facilitates neutrophil exocytosis to release myeloperoxidase and lysozyme. After respiratory virus invasion, PF4 is capable of stimulating antigen-presenting cells (APCs) to induce the proliferation of lymphocytes and the cytotoxic activity of NK cells. PF4 plays a critical role in the clearance of viruses, as PF4 KO mice show diminished viral clearance from the lung as compared to wild-type mice [21]. This feature is consistent with the reduced innate immunity observed in the PF4 KO mice during early infections.

*Plasmodium falciparum* infection leads to malaria, an infection that kills ~half million people worldwide annually. During *Plasmodium falciparum* infection, platelets release PF4 to kill intraerythrocytic *P. falciparum* parasites via the Duffy-antigen receptor (Fy/DARC) on erythrocytes [22]. During HIV-1 infections, activated platelets also produce PF4, which acts as an anti-viral cytokine. The oligomeric status of PF4 influences whether PF4 inhibits or enhances HIV infection. When PF4 exists predominantly in a monomeric state, it binds to the HIV-1 envelope protein gp120 and inhibits HIV-1 attachment to the cell surface, thus reducing HIV-1 entry into CD4+ T cells [23]. HIV-1-infected patients with low serum levels of PF4 tend to have more advanced clinical outcomes. As its concentration increases, PF4 tends to form tetramers or higher-ordered forms, which enhance HIV-1 infection in vitro. Soluble glycosaminoglycans (GAGs) can prevent the tetrameric PF4 from enhancing virus infection [24], indicating that interacting with cell surface GAGs is required by the tetrameric PF4 to help HIV infection. PF4 may also be involved in host responses to coronavirus infection. Protein–protein interactions (PPI) and gene co-expression data indicate that PF4 is among cytokines produced by host innate immunity in response to the S-glycoprotein of MERS-CoV [25].

While there are clearly anti-infective activities of PF4, the PF4 species itself may also contribute to pathogenic infections. As a pro-inflammatory cytokine, PF4 is related to the pathogenesis of cerebral malaria (CM), a serious complication of *P. falciparum infection*. This may occur after the disruption of the blood–brain barrier [26].

## 4. Structural Basis for PF4 Function: Binding Activity of PF4 to Polysaccharides

Heparin is a naturally occurring GAG and has been clinically used as an anticoagulant in the treatment of heart attacks and unstable angina, and most recently in the clinical care of COVID-19 patients [27]. GAGs are long linear polysaccharides consisting of repeating disaccharide units. This feature is distinct from N- or O-linked glycans, which are branched polysaccharides. The repeating unit of GAGs typically consists of a uronic acid (or galactose) and an amino sugar (N-acetylglucosamine or N-acetylgalactosamine). The acidic sugar residues and sulphate groups account for the high negative charge of GAGs.

**Figure 1:**
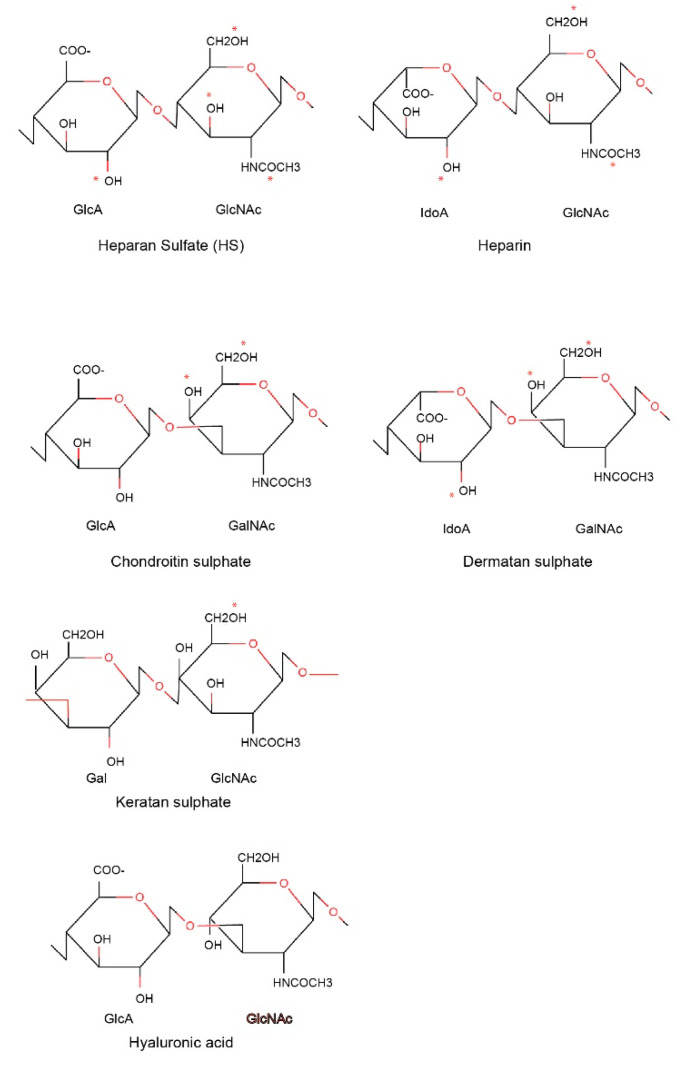
Structure of glycosaminoglycan chains. The repeating disaccharides in different types of glycosaminoglycan chains are presented. The different sulphation positions in each glycosaminoglycan (GAG) are marked by * in red.

Based on core disaccharide structures, GAGs are classified into four groups (Figure 1):

- Heparin/heparan sulfate;
- Chondroitin sulfate/dermatan sulfate (CSGAGs);
- Keratan sulfate;
- Hyaluronic acid.

The difference between heparin and heparan sulfate moieties relates to cellular location. Heparan sulfate polysaccharides exist ubiquitously on the cell surface and extracellular matrix, whereas heparin is present within mast cells and can be considered as a more sulfated variant of heparan sulfate. Heparin/heparan sulfates are synthesized in the Golgi apparatus and attached by glycosyltransferases to protein cores via O-linked glycosylations. Proteins carrying GAGs are called Proteoglycans (PGs). One of the platelet proteins that heparin/heparin are abundantly attached to is serglycin, an intracellular proteoglycan found in platelet and other hematopoietic cells, as well as endothelial cells. Differences exist in the GAG attachment on serglycin in different tissues, with heparin and chondroitin sulfate as the preferred GAGs in connective tissue mast cells and mucosal mast cells/activated macrophages, respectively. Chondroitin sulfate/dermatan sulfate are also synthesized in the Golgi apparatus and attached to protein cores via O-linked glycosylations.

As reported by Kolset et al., the multimeric forms of PF4 are the major platelet-derived proteins that are bound to serglycin [28]. PF4 has a very high affinity for heparin (K_d_: ~30 nM), while its affinity for heparin sulfate and serglycin is about 10-fold less. Like other platelet-derived chemokines, such as RANTES and pro-platelet basic protein (PPBP), PF4 has a positively charged antimicrobial peptide (AMP) domain. With its positively charged surface, PF4 is also able to bind to heparin, which has the highest negative charge density of all known natural molecules due to its heavily sulfated glycosaminoglycans.

## 5. Heparin-Induced Thrombocytopenia (HIT)

As a widely used anti-coagulant during invasive vascular surgery, heparin therapy (in 1–5% of individuals so treated) can cause a complication termed heparin-induced thrombocytopenia (HIT) [29,30]. In patients with HIT, antibodies against PF4/heparin complexes induce thrombocytopenia and thrombosis, even under situations when the platelet counts are low [31]. This is a life- threatening immune-mediated disorder that typically occurs 4–10 days after exposure to heparin, and is different from so called “Type 1 HIT”, in which thrombocytopenia occurs within the first 2 days after exposure to heparin, but platelet count returns to normal with continued heparin therapy.

To identify the risk for HIT in patients who are currently or were recently treated with heparin- derived agents, a 4 T’s -scoring system has been used [32,33]. This scoring system evaluates the following clinical features: the degree of Thrombocytopenia, the Timing of platelet count fall, the associated Thrombosis, and the concurrent presence of other potential etiologies of Thrombocytopenia. For patients with intermediate or high-risk 4T scores, further laboratory evaluation for HIT or switching to a non-heparin derived anti-coagulant is recommended. The diagnosis can be essentially confirmed if the 4 T’s score is ≥4 and the HIT IgG-specific antibody is positive.

To reduce the risk of HIT, the use of low-molecular weight heparin (LMWH) instead of unfractionated heparin is suggested [34]. However, there are still reports of HIT after the use of LMWH, such as Enoxaparin [35]. Alternative, a homogenous synthetic anticoagulant fondaparinux, which is a modified pentasaccharide heparin, can also be used. Although fondaparinux can still form complexes with PF4 [36], the complexes are poorly recognized by HIT antibodies [37]. Therefore, fondaparinux renders lower risk for HIT. Because of this, fondaparinux has been used in HIT patients complicated with or without thrombosis [38,39,40,41]. However, fondaparinux is not an ideal treatment for HIT. Low-dose fondaparinux does not necessarily prevent thrombotic complications of HIT [42]. Furthermore, it is reported that a patient who underwent aortic stent graft placement developed thrombocytopenia after fondaparinux was used for anticoagulation [43]. In stroke patients, administration of fondaparinux led to further reduction in platelet counts. Heparin-induced platetet activation assay (HIPA) showed platelet activation in the presence of both heparin and fondaparinux, although IgG-PF4/heparin antibodies were not detected [44]. To rescue patients from fondaparinux- cross reactive or fondaparinux-refractory HIT, high-dose intravenous immunoglobulin (IVIG) was clinically used [45]. Switching blood thinners to rivaroxaban is considered safe and effective for the management of clinically suspected HIT [46].

Heparin, PF4, and the PF4 antibody forms HIT immune complexes that induce the crosslinking of Fc-receptors on platelets, neutrophils and monocytes and lead to the production of pro-inflammatory and procoagulant cytokines, such as PF4, tissue factor (TF) and thrombin [47,48]. For example, binding of the HIT immune complexes to human monocytes induces TF expression on peripheral blood mononuclear cells (PBMCs) and monocytes [49]. TF is the high-affinity receptor and cofactor for Factor VII/VIIa (FVII/F VIIa), and the TF-FVIIa complex is the primary initiator of blood coagulation cascade that generates coagulation proteases, such as FXa, and thrombin [50]. Although the FcγRIIA on monocytes is critical for the HIT immune complexes to bind monocytes [51], Cines and colleagues have recently pointed out that FcRn, which is commonly involved in the recycling of IgGs, augments the induction of TF activity by the HIT immune complexes [52].

Since HIT is an immune disease, and patients may have elevated pro-inflammatory cytokines, cyclophosphamide may help overcome certain features of cytokine storm during HIT. For example, a patient with severe lupus nephritis (SLE) developed HIT during treatment and the thrombin inhibitor argatroban was used for thrombosis prophylaxis. Due to decreases in oxygen saturation and blood pressure, the patient with congestive heart failure was admitted to the intensive care unit (ICU). The patient received medical support for his circulation and was treated with antibiotics for microbial infection. The patient’s myocardial damage, however, was believed to be caused by cytokine storm. After treatment with methylprednisolone (mPSL) pulse therapy, the patient showed stable vital signs and was discharged from ICU. His thrombocytopenia persisted and hemoptysis occurred two days later. The patient was found to have alveolar hemorrhage. He was admitted to the ICU again due to decreased oxygen saturation, and started on ventilator management with endotracheal intubation. Eventually the patient responded to cyclophosphamide, an inhibitor to suppress the immune system in general to control SLE activity. In three days, the patient displayed increased platelet count, and was released from mechanical ventilation and discharged from the ICU [53].

## 6. Structural Basis for Heparin-Induced Thrombocytopenia (HIT)

PF4 is a cationic monomeric protein with a propensity to form tetramers that assemble into oligomers in the presence of polyanions [54,55,56]. To form the immune complex that induces HIT, PF4 first binds heparin or other polyanions to obtain a structure that has much higher affinity and avidity for a set of HIT antibodies [57]. Through the Fc of recruited HIT antibodies, the micron-sized immune complex can cross-link cell-surface Fc receptors and activate platelets and other types of immune cells. The activated platelets have procoagulant activity, which leads to a pathology contrary to the expected activity of the anti-coagulant activity of heparin [58].

Not all PF4 antibodies are pathogenic and lead to HIT [59,60,61,62]. Pathogenic HIT antibodies are known to bind PF4 in the presence of heparin. However, current clinical tests are not effective to determine whether the PF4 antibodies are pathogenic or non-pathogenic. Arepally and colleagues identified that the murine monoclonal antibody KKO forms PF4/heparin complexes and causes heparin-induced thrombosis and thrombocytopenia in a murine model, thus classifying KKO as a pathogenic antibody [59]. Human HIT antibodies compete with KKO for binding to PF4/heparin, and KKO augments the formation of pathogenic immune complexes [63].

**Figure 2:**
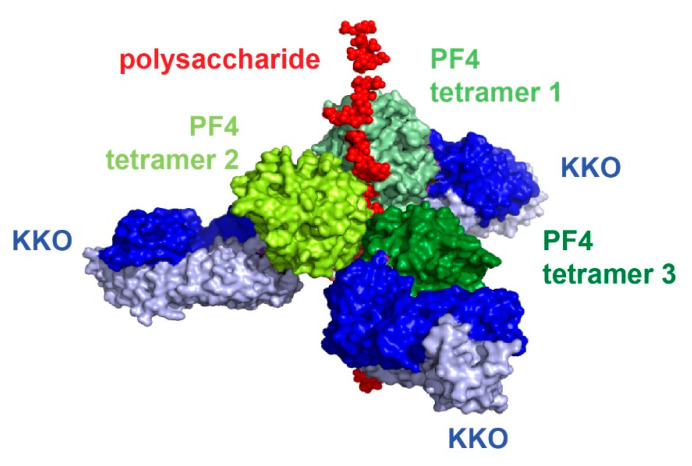
Current understanding of the structure of the HIT immune complex.

To understand how PF4 structurally interacts with heparin and pathogenic HIT antibodies, our laboratory successfully crystallized PF4 in complex with fondaparinux [64]. More importantly, we also obtained the crystal structure of PF4 tetramer with the pathogenic antibody KKO [64]. These two structures enabled our laboratory to understand the structure of the HIT immune complex (Figure 2, [64]) and, along with the solution of other crystal complexes, determine the atomic pathway that leads to HIT in vivo. PF4 tetramers cluster around a semi-rigid linear heparin chain that resides in the middle of the complex. Multiple HIT antibodies KKO (blue) bind to the outer surface of PF4 tetramers, ultimately assembling into the ultra-large immune complexes (ULICs) that mediate cell activation. Heparin and HIT antibodies operate in a collaborative manner to stabilize the PF4 tetramers and the resultant ULICs [64].

## 7. Coagulation Dysfnction in COVID-19 and the Risk of HIT

Thrombocytopenia is a common clinical feature of viral infections, especially for RNA viruses (e.g., HIV-1, HCV, dengue) [65,66]. The occurrence of thrombocytopenia can be caused by platelet activation and platelet-monocyte aggregate formation after viral infection [67]. In COVID-19 patients, elevated concentrations of inflammatory cytokines have been observed [68]. The release of excessive cytokines damages endothelial cells and disturbs their function, leading to problems with coagulation dysfunction and microvascular disease [69,70]. Thrombosis within the brain can lead to strokes, and clots in the lungs can reduce blood flow into the lungs and limit the usefulness of ventilators.

Being caused by the infection of severe acute respiratory syndrome coronavirus 2 (SARS-CoV-2), COVID-19 was initially defined as a lung disease. However, due to the inflammatory cytokine storms induced by SARS-CoV-2 infection [71,72,73], the coagulation pathway can be activated in severe COVID-19 patients and cause excessive consumption of coagulation factors and platelets. Subsequently, coagulation dysfunction can occur in a certain percentage of patients, and thrombosis is one of the major causes of death in patients with severe COVID-19 [74,75,76,77]. In one study from China involving 183 COVID-19 patients, non-survivors had significantly higher D-dimer and fibrin degradation product (FDP) levels. The normal range of D-dimer is under 0.5 μg/mL, but the average D-dimer levels in survivors and non-survivors were 0.61 and 2.12 μg/mL, respectively. During their hospital stay, 71.4% of non-survivors, vs. 0.6% of survivors met the criteria of disseminated intravascular coagulation [75]. In French studies, 23~30% of COVID-19 patients were found by computed tomography (CT) to have pulmonary embolisms [78,79]. These patients also had higher D-dimer levels and were more likely to be in the intensive care unit.

In a prospective study of hospitalized COVID-19 patients in non-intensive care units, 156 patients with D-dimer > 1 μg/mL were screened for asymptomatic DVT with complete compression doppler ultrasound (CCUS). A total of 23 patients (14.7%) were identified to have DVT. Patients with DVT had higher median D-dimer levels: 4.527 μg/mL vs. 2.05 μg/mL (*p* < 0.001). D-dimer levels > 1.57 μg/mL were associated with asymptomatic DVT (OR 9.1; CI 95% 1.1–70.1) [80]. When autopsies were performed in a German hospital on the first 12 consecutive COVID-19-positive deaths, deep venous thrombosis was found in seven patients (58%). None of these patients were suspected to have venous thromboembolism before death. In addition, pulmonary embolism was identified as the direct cause of death in four patients [81]. In another autopsy study of 11 deceased COVID-19 patients, thrombosis of small and mid-sized pulmonary arteries was found in various degrees in all these patients and was associated with infarction in eight patients and bronchopneumonia in six patients [74]. Again, none of these patients had clinical evidence of venous thromboembolism before death, although 10 of the 11 patients had already received prophylactic anticoagulant therapy.

Since elevated D-dimer levels are often found in patients with clotting problems, severe COVID-19 patients with elevated D-dimer levels are treated with unfractionated heparin or LMWH as an anticoagulation to reduce mortality (Table 1) [82,83,84,85,86,87]. However, the current anticoagulation options have limited effect on thrombosis in general COVID-19 patients, and some of the observed effects were not significant [88,89,90,91]. In a Dutch study of COVID-19 patients, all 184 ICU patients received at least standard doses thromboprophylaxis, but thrombosis still occured in 31% patients, including 27% venous thromboembolism (VTE) and 3.7% arterial thrombotic events [92]. Pulmonary embolism was the most frequent thrombotic complication (81%). The dosage of anticoagulation agents is also important for the management of COVID-19 patients. It is possible that some patients benefited most from the use of anti-coagulants. In a retrospective review of 245 COVID-19 patients admitted to the ICU requiring mechanical ventilation at Mount Sinai Hospital, patients treated with therapeutic anticoagulation for a minimum of 5 days had a 79% reduction in death when compared with those treated with prophylactic dose [27]. Although a slight trend towards increased bleeding complications was observed in the therapeutic anticoagulation group, the effect was not statistically significant. It is thought that the benefit of initiating therapeutic anticoagulation in intubated COVID19 patients outweighs the risk of bleeding.

Thrombocytopenia is another problem in COVID-19 patients [93,94]. In one study from China, which involved 1099 COVID-19 patients, thrombocytopenia was present in 31.6% of non-severe patients, but was observed in 57.7% of patients with severe disease [93]. While the coagulation in COVID-19 is related to the host response to SARS-CoV-2 infection, thrombocytopenia can be HIT, caused by the use of heparin [95]. In a retrospective cohort analysis of 652 hospitalized patients with COVID-19 at Beth Israel Deaconess Medical Center, 88 patients who received at least 5 days of UFH were selected to check if HIT occurred. Eight patients had suspected HIT, and five patients tested positive for HIT by latex immunoassay. All five patients were treated with direct thrombin inhibitors after HIT antibody test (four with argatroban, one with bivalirudin). While three patients developed major hemorrhagic events and died after progressive respiratory failure, only one patient suffered acute cerebrovascular infarct as well as extensive areas of splenic infarction [96].

There are abundant reports of thrombocytopenia in severe COVID-19 patients. In a study of 61 critical COVID-19 patients admitted into intensive care unit (ICU) and 93 severe non-ICU patients in the Huoshenshan Hospital (Wuhan, China), 41% of ICU patients (25/61) had severe thrombocytopenia with a platelet count less than 50 × 10^9^/L. All except one (24/25) had a fatal outcome. 52.2% of non-survivors (24/46) had severe thrombocytopenia, compared to 6.7% (1/15) in survivors. High levels of anti-Heparin-PF4 antibodies were observed in most of ICU patients [95]. Riker et al. reported the cases of thrombocytopenia with anti-PF4 antibodies among 16 intubated COVID-19 patients with ARDS. All three patients had evidence of thrombosis (pulmonary embolism, upper extremity venous thromboses, and skin necrosis, respectively). One case was confirmed as HIT by the serotonin release assay [97].

In addition to the use of anticoagulation agents for potential coagulation dysfunction in COVID-19, some medical care procedures also require the use of heparin. For critically ill COVID-19 patients, extracorporeal membrane oxygenation (ECMO) is used to replace the function of the lungs and heart. ECMO pumps and oxygenates the patient’s blood outside the body, for which unfractionated heparin is frequently administered to patients to prevent coagulation. This also exposes these severe or critically ill patients to a high risk of HIT. In a study of patients with severe heart failure, out of 57 adult patients who underwent an ECMO for at least 5 days, 29 patients (50%) were positive for PF4-specific Abs. HIT was suspected in two patients with ECMO dysfunction and unexpected platelet count decrease after day 5. These two patients also had high levels of PF4-specific IgG levels. Eventually, HIT was confirmed in both individuals by a serotonin release assay [98].

Much more frequent thrombocytopenia episodes are also observed among those who underwent continuous renal replacement therapy (CRRT), a procedure commonly used in the intensive care unit for correction of metabolic acidosis and removal of cytokines in critically ill COVID-19 patients. Unfractionated heparin is the most commonly used anticoagulant to maintain circuit patency during CRRT [99]. In one study, most of the 16 CRRT patients (13/16) experienced a sharp decrease in platelets to less than 50 × 10^9^/L. In comparison, only 26.7% of non-CRRT ICU patients (12/45), and 1.1% of non-ICU patients (1/93) showed such a decrease in platelets [95]. The high risk of thrombocytopenia in CRRT patients suggests that heparin use in CRRT might contribute to the high mortality of critically ill COVID-19 patients.

Thus, for a severe case of COVID-19, if the platelet count is decreased by >50% from the basal value and there are signs of arteriovenous thrombosis after an administration of heparin, patients should be evaluated for HIT. For COVID-19 patients using ECMO support, it is recommended that patients are monitored for HIT. If the ECMO circuit is frequently abnormal and platelets progressively decrease, and if there are high levels of PF4-specific IgG antibodies, HIT should be considered.

It was also noticed that HIT antibodies and a progressive decrease in platelets occurred in heparin-naïve patients (i.e., before CRRT or other heparin exposure), and even in non-ICU patients, suggesting that a spontaneous HIT might occur in COVID-19 patients, probably related to the formation of PF4 tetrameric complexes during viral or secondary bacterial infection. In COVID-19 patients, the serum concentrations of HIT antibodies are largely correlated to C3a levels. Since complement activation can induce the release of heparin from master cells, spontaneous HIT in COVID-19 may be related to the complement activation that was reported for IgM [100].

## 8. Potential Use of a Humanized PF4 Antibody to Prevent HIT

**Figure 3:**
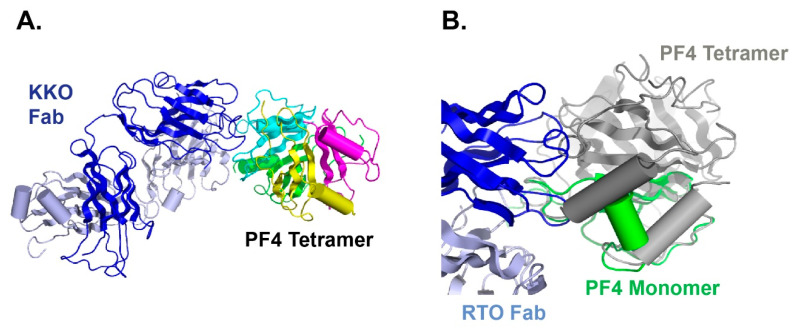
RTO binding to PF4 monomer impedes PF4 from forming tetramers, thus prevent PF4 tetramer-based immune complex formation, which underlies the pathogenesis of HIT. (**A**) HIT-like antibody KKO (blue) binds to PF4 tetramers (green, yellow, cyan and magenta; (**B**) blocking antibody RTO (blue) binds to PF4 monomer (green) and prevents tetramer (gray) formation.

As we mentioned before, not all PF4 antibodies are pathogenic, and this actually creates problems in HIT diagnosis. While KKO can cause HIT, an isotype matched anti-PF4 antibody RTO binds PF4 but does not generate pathogenic complexes [101]. KKO promotes the oligomerization of PF4, while RTO only binds to the PF4 monomer and prevents it from oligomerization [63]. We have also solved the crystal structure of the PF4 monomer in complex with RTO [64]. Our data indicated that RTO binds to the PF4 monomer and prevents it from forming tetramers (Figure 3), a critical structure that is required for HIT antibody binding and HIT immune complex formation. Since PF4 exists in a dynamic equilibrium between monomers and tetramers, stabilizing monomer populations of PF4 proteins will promote the dissociation of PF4 tetramers, thereby preventing the subsequent development of PF4 tetramer-based pathogenic immune complexes [64].

**Figure 4:**
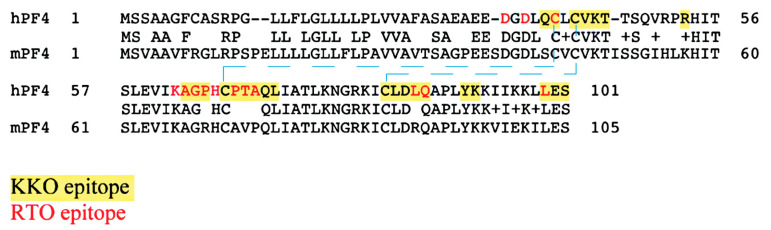
Comparison of human PF4 (hPF4) and mouse PF4 (mPF4) sequences, showing the overlap of KKO and RTO epitopes. Since KKO preferentially binds to PF4 tetramer, its epitopes are contributed by amino acid residues from different chains within a PF4 tetramer. Dash lines depict the internal disulfide bonds.

KKO and RTO have shared but not identical epitopes [61,102] (Figure 4). To verify that RTO can block the pathogenic activity of KKO, we performed both in vitro and in vivo assays [64]. When platelet-rich plasma is incubated with PF4 and KKO, platelet activation and aggregation can be observed by measuring light transmission using a dual-channel lumi-aggregometer. However, when platelet-rich plasma is pre-incubated with RTO, KKO-induced platelet aggregation is prevented. The in vivo activity is demonstrated with the cremaster laser injury model performed on transgenic male C57BL mice that are deficient in mouse PF4 but express human PF4 and human FcγRIIA26 [103,104]. After focal arterial injury, circulating platelets can be observed with a fluorescently labeled CD41 antibody. In the presence of KKO, the formation of thrombus is observed. If mice are pre-treated with RTO, the effect of KKO-induced thrombosis is completely inhibited [64]. Our data have shown that RTO is a blocking antibody, as it blocks the in vitro and in vivo platelet-activating capacity of KKO.

RTO is a mouse monoclonal antibody. With current data suggesting that RTO, as an HIT-blocking antibody, can be developed as a non-anticoagulant intervention in HIT, RTO needs to be humanized first before the antibody can be tested in clinical trial [105,106]. Recently, we have humanized the RTO antibody and are planning for pre-clinical testing and, ultimately, clinical application.

## 9. Conclusions

COVID-19 is not just a lung disease, but also a vascular and coagulation disease that is related to immune response to virus infection. Therapies targeting overreactive immune response and cytokine release storm are under clinical trials [68,107]. As coagulation abnormalities occur frequently in COVID-19 patients, the clinical use of heparin to prevent coagulation may lead to HIT. HIT symptoms and clinical signs should be monitored, especially in patients prone to developing this problem.

Although anticoagulants such as argatroban/bivalirudin may be administered instead of heparin to avoid HIT, they are not perfect solutions and issues with reversing the activity of argatroban/bivalirudin should be considered [108]. HIT-blocking antibodies should be investigated for potential clinical use to prevent fatal outcomes after HIT in COVID-19-infected individuals.

## Figures and Tables

**Table 1:** Use of anticoagulants and outcomes in COVID-19 patients. CHD: chronic heart disease; aRR: adjust risk ratio; HR: Hazard ratio; OR: odd ration; LMWH: Low molecule weight heparin.

Patient Number	Patient Condition	Anti-Coagulation	Outcome	Ref.
2773	hospitalized	therapeutic anticoagulation	For patients who required mechanical ventilation (*n* = 395), in-hospital mortality was lower in those treated with anticoagulation (29.1% vs. 62.7%); no difference in general population	[84]
2075	hospitalized	Heparin	Lower mortality in patients who used Heparin (OR 0.55, 95% CI 0.37–0.82, *p* = 0.003)	[85]
1716		therapeutic anticoagulation	Subjects receiving new therapeutic anticoagulation, especially for those in the absence of other indications, were more likely to die (OR 5.93; 95% CI 3.71–9.47). Continuation of outpatient prescribed anticoagulant was not associated with improved clinical outcomes.	[91]
449	severe	mainly LMWH	In patients with D-dimer >6-fold of upper limit of normal, 28-day mortality was lower in heparin users than nonusers (32.8% vs. 52.4%, *p* = 0.017).	[83]
374	hospitalized	therapeutic vs. prophylatic	higher in-hospital mortality in patients receiving preemptive therapeutic anticoagulation (aRR: 2.3, 95% CI = 1.0, 4.9; *p* = 0.04)	[90]
245	ICU	therapeutic vs. prophylatic	79% reduction in death with therapeutic dose	[27]
184	ICU	thromboprophylaxis	thrombosis rate: 31%	[92]
115	hospitalized	therapeutic anticoagulation	Lower mortality in patients with anticoagulation (OR 0.055, 95% Cl 0.008–0.386, *p* = 0.03)	[87]
101	Nursing Home Residents		Only a trend of lower mortality in patients with anticoagulation (OR 0.89, 95% Cl 0.41–1.95)	[89]
70	elderly patients with interstitial pneumonia and CHD	direct oral anticoagulants	Lower mortality in patients with anticoagulation (HR 0.38, 95% Cl 0.17–0.58, *p* = 0.01)	[86]
